# Pigmentation plasticity enhances crypsis in larval newts: associated metabolic cost and background choice behaviour

**DOI:** 10.1038/srep39739

**Published:** 2017-01-04

**Authors:** Nuria Polo-Cavia, Ivan Gomez-Mestre

**Affiliations:** 1 Department of Biology, Universidad Autónoma de Madrid, Ciudad Universitaria de Cantoblanco, 28049 Madrid, Spain; 2 Ecology, Evolution, and Development Group, Doñana Biological Station, CSIC. E-41092 Seville, Spain

## Abstract

In heterogeneous environments, the capacity for colour change can be a valuable adaptation enhancing crypsis against predators. Alternatively, organisms might achieve concealment by evolving preferences for backgrounds that match their visual traits, thus avoiding the costs of plasticity. Here we examined the degree of plasticity in pigmentation of newt larvae (*Lissotriton boscai*) in relation to predation risk. Furthermore, we tested for associated metabolic costs and pigmentation-dependent background choice behaviour. Newt larvae expressed substantial changes in pigmentation so that light, high-reflecting environment induced depigmentation whereas dark, low-reflecting environment induced pigmentation in just three days of exposure. Induced pigmentation was completely reversible upon switching microhabitats. Predator cues, however, did not enhance cryptic phenotypes, suggesting that environmental albedo induces changes in pigmentation improving concealment regardless of the perceived predation risk. Metabolic rate was higher in heavily pigmented individuals from dark environments, indicating a high energetic requirement of pigmentation that could impose a constraint to larval camouflage in dim habitats. Finally, we found partial evidence for larvae selecting backgrounds matching their induced phenotypes. However, in the presence of predator cues, larvae increased the time spent in light environments, which may reflect a escape response towards shallow waters rather than an attempt at increasing crypsis.

Concealment from predators is of capital importance in an organism’s life, given that predation is one of the strongest selective pressures in nature. As such, camouflage is a widespread behavioural tactic employed by animals in order to reduce the risk of being detected or recognized^1^,^2^,^3^. Camouflage strategies are diverse, including background matching through crypsis^4^,^5^,^6^, disruptive coloration promoting misidentification of the outlines of the body^7^,^8^,^9^,^10^, or even masquerade (*i.e.*, resemblance of an organism to an inedible object)^11^,^12^,^13^. Although defensive adaptations may target sensory systems other than vision^14^, most remarkable examples involve visual camouflage and cryptic coloration.

While many animals have evolved constitutive colour patterns matching their habitats as key adaptions, organisms may often face spatial or temporal environmental heterogeneity so that a single pigmentation pattern may not provide crypsis across all environmental conditions^15^. One way to maximize crypsis in those cases is evolving a coloration constituting a compromise between the requirements of the differing microhabitats^16^,^17^,^18^,^19^. Alternatively, opposing selection pressures can generate a prey species to evolve two or more distinct morphs, each one adapted to a different part of the environment (i.e., polymorphic crypsis)^20^. Apart from promoting these constitutive adaptions, selection is expected to favour the capacity to plastically alter coloration depending on the surrounding conditions^21^. As it is also true for other plastic traits, colour change may be energetically costly, as it requires cell migration and synthesis or recruitment of pigments, and may even compromise the immune system through reallocation of resources to pigment production^22^,^23^,^24^,^25^. In rapidly changing environments, alterations in the pigmentation pattern must be reversible and promptly elicited to be effective in the short-term^26^. However, if such alterations are costly, we hypothesize that organisms would more readily trigger colour changes when the perceived risk of predation is higher, so that they would be more prone to pay the cost of shifting pigmentation as the environment changes in the presence of predators than in predator-free environments. Moreover, if changes in pigmentation entail costs and organisms have the chance to move across dissimilar habitat patches, they might have evolved preferences for patches in which their colour patterns grant better crypsis, rather than incurring the costs of altering their pigmentation. Also, prey might have evolved background matching preferences to reduce detectability if their ability to change colour is not quick enough to match the pace of environmental change. Here we tested these ideas about the interplay of pigmentation plasticity, costs of pigment production, and habitat choice using amphibian larvae as study system.

Many amphibians rely on cryptic coloration as their first line of defence^27^. By achieving colour patterns resembling habitat features, both anurans and salamanders avoid detection from visual predators, thereby improving their fitness^28^,^29^,^30^,^31^. In addition to constitutive adaptations, amphibians can increase concealment by expressing plasticity in skin coloration. In response to environmental stimuli such as background colour, light intensity, temperature, humidity or stress, melanosomes (*i.e.*, the light absorbing organelles) can be reallocated in the melanophores (*i.e.*, a type of pigment cells within the dermal chromatophore unit), resulting in a lightening (pigment aggregation) or darkening (pigment dispersion) of the skin^32^,^33^,^34^,^35^,^36^,^37^. Although these colour shifts are not instantaneous as those exhibited by chameleons, flatfish or cephalopods^38^,^39^,^40^, both larvae and adults of many amphibian species benefit from relatively rapid (*i.e.*, within minutes) as well as more long-term (i.e., several weeks) colour change, which can be effective in hampering predator detection^3^,^41^.

Although the ability of amphibians to adjust skin coloration may result advantageous in achieving crypsis, individuals may incur costs and trade-offs associated to colour change^23^,^24^,^26^. This may represent, for instance, an important constraint for larvae to conceal in dark, low-reflecting environments, since rearranging pigment organelles in the dermis (or even increasing the number of melanosomes and chromatophores) requires energy expenditure, which may result in competing demands with other physiological processes and growth^22^,^23^. An accurate assessment of predatory threats seems therefore essential for amphibian larvae to maximize the benefits of pigmentation plasticity. In several anuran species, larvae respond to the presence of predators by increasing tail coloration^42^,^43^,^44^,^45^. This predator-induced phenotype is interpreted as a defensive strategy to deflect attacks of predators away from critical parts of the body^9^,^46^,^47^. Antipredator responses can be so fine-tuned to the risk of predation that the same amphibian species may markedly increase coloration at the distal portion of the tail in the presence of ambush predators, or reduce pigmentation altogether in the presence of fish, which are much more visually guided predators^48^.

Because background matching effectively reduces predation^17^,^49^,^50^, a common assumption is that prey have been selected to prefer appropriate habitats matching their visual aspect. Although few studies have found empirical support for such behaviour^51^,^52^,^53^,^54^,^55^,^56^, the idea that prey recognize and show preferences for backgrounds that confer greater crypsis against predators has been often presumed, also in amphibian larvae^57^,^58^.

The vast majority of studies on amphibian camouflage have focused on anurans^28^,^33^,^34^,^37^,^59^,^60^ (see ref. 61 for an extensive review), whereas the adaptive value of cryptic coloration and behaviour of salamanders –aside from their aposematic traits^62^,^63^,^64^– has received considerably less investigation. We analysed the expression of phenotypic plasticity in skin pigmentation of larvae of the Iberian newt, *Lissotriton boscai*, in response to different albedo environments and the presence of chemical cues from a common predator: nymphs of the dragonfly *Anax imperator*. We tested the following hypotheses: (1) *L. boscai* larvae have the ability to adjust skin pigmentation to different reflecting microhabitats, (2) such changes in pigmentation are modulated by perceived risk of predation, (3) induced pigmentation changes are reversible, (4) there are metabolic costs associated to increased skin pigmentation, and (5) background choice behaviour is conditional upon individual level of pigmentation. We expected larvae to gradually darken or lighten depending on environmental albedo, in order to match their backgrounds and improve concealment against potential predators. If altering pigmentation entailed substantial metabolic costs, we expected exposure to predator cues to make larval newts more prone to pay such costs in order to achieve fine-tuned background colour matching.

## Results

### Skin pigmentation

**Figure 1:**
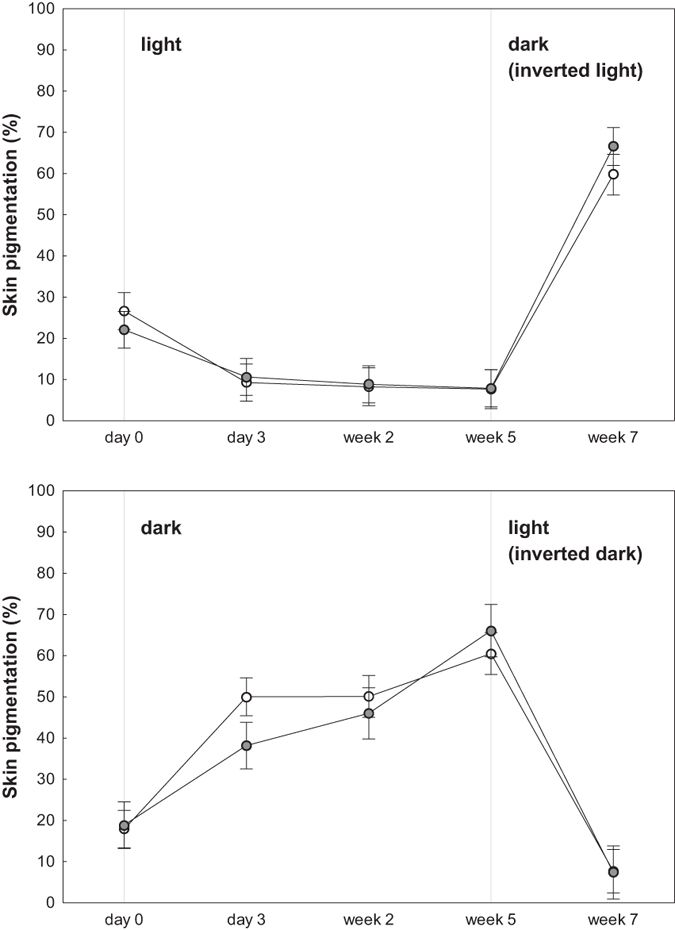
Mean ± SE of changes in skin pigmentation of larval newts induced by environmental albedo. From day 0 to week 5: induced changes under light (upper panel) and dark (bottom panel) microhabitat conditions. From week 5 to week 7: reverted changes after inverting microhabitat conditions. Open circles represent larvae in a predator-free environment; solid circles represent larvae exposed to predator cues.

Environmental albedo induced significant changes in skin pigmentation of larval newts throughout the first five weeks of the experiment. Skin pigmentation of larvae in light, high-reflecting microhabitats decreased by 68% on average, whereas it increased by 347% on average in dark, low-reflecting microhabitats (F_3,56_ = 75.81, p < 0.0001; Fig. 1). At the beginning of the experiment (day 0), skin pigmentation of larvae in light and dark microhabitat conditions was similar (p = 0.76; Fig. 1). Intra-individual changes in skin pigmentation started to be noticeable as early as 3 days after the experiment began, both for light and dark conditions (p < 0.0001 in both cases). From day 3 to week 2, skin pigmentation of larvae in light conditions decreased even further (p = 0.04), but pigmentation of larvae in dark conditions remained invariant (p = 0.99). From week 2 to week 5, skin pigmentation of larvae did not vary within microhabitat (p = 0.21 and p = 0.12 for light and dark conditions, respectively). Differences in skin pigmentation between the two microhabitat treatments were clear at day 3, and continued to be significant during the rest of the experiment (p < 0.0001 in all cases) (Fig. 1). Exposure to predator cues had no significant effect on changes in skin pigmentation (F_3,56_ = 0.45, p = 0.72), and did not interact with microhabitat conditions (F_3,56_ = 0.58, p = 0.63).

### Reversibility

Changes in skin pigmentation of larval newts induced by microhabitat conditions were reverted from week 5 to week 7 (F_1,37_ = 915.88, p < 0.0001; Fig. 1). Thus, after inverting microhabitat conditions, larvae in both light and dark treatments adjusted skin pigmentation accordingly: larvae moved from light to dark conditions drastically increased pigmentation from week 5 to week 7 (p = 0.0002). Likewise, larvae moved from dark into light conditions markedly reduced their pigmentation during the same period of time (p = 0.0002) (Fig. 1). Skin pigmentation of larvae between the two microhabitats differed clearly on week 7 (p = 0.0002). Neither the presence of predator cues nor its interaction with microhabitat conditions affected the reversibility of skin pigmentation of larval newts (F_1,37_ = 0.47, p = 0.5 and F_1,37_ = 0.06, p = 0.81 respectively).

### Metabolic costs

**Figure 2:**
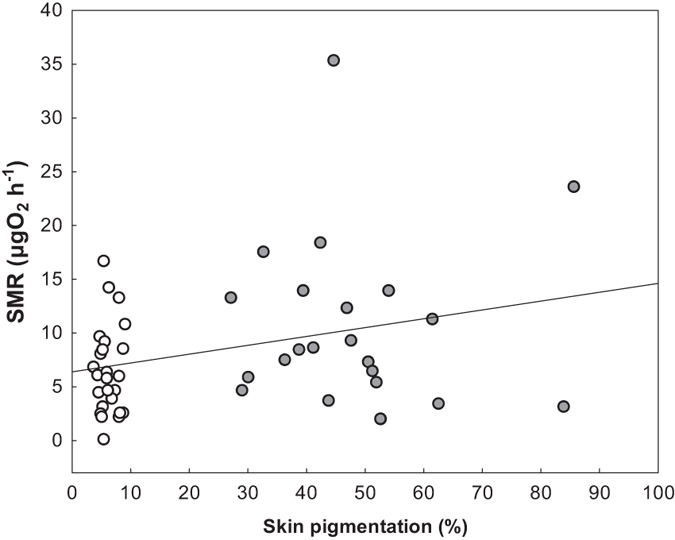
Relationship between skin pigmentation and standard metabolic rate (SMR) of larval newts, after two weeks of experiment. Open circles represent unpigmented larvae subjected to light microhabitat conditions; solid circles represent heavily pigmented larvae subjected to dark microhabitat conditions.

To assess the potential metabolic cost associated to skin pigmentation or depigmentation of larval newts, we measured standard metabolic rate (SMR) of larvae exposed to different microhabitat treatments. SMR of larval newts differed between light and dark conditions (F_1,45_ = 5.45, p = 0.02). After two weeks of experiment, larvae in dark conditions showed 64% higher rates of oxygen consumption (mean ± SE: 10.73 ± 1.31 μg O_2_ h^−1^) than larvae in light conditions (6.55 ± 1.22 μg O_2_ h^−1^). Larvae exposed to predator cues showed similar SMR than larvae exposed to clean water, and exposure to predator cues neither affected SMR of larvae within each microhabitat treatment (i.e., predator cue treatment and its interaction with microhabitat had no significant effect and were dropped from the model). When included as a covariate, percentage of skin pigmentation had no significant effect on SMR. However, when the collinear effect of microhabitat was not included in the model, there was a significant positive correlation between percentage of skin pigmentation and SMR, with more pigmented larvae showing higher rates of oxygen consumption (r = 0.32, F_1,45_ = 5.15, p = 0.03; Fig. 2).

### Background choice behaviour

**Figure 3:**
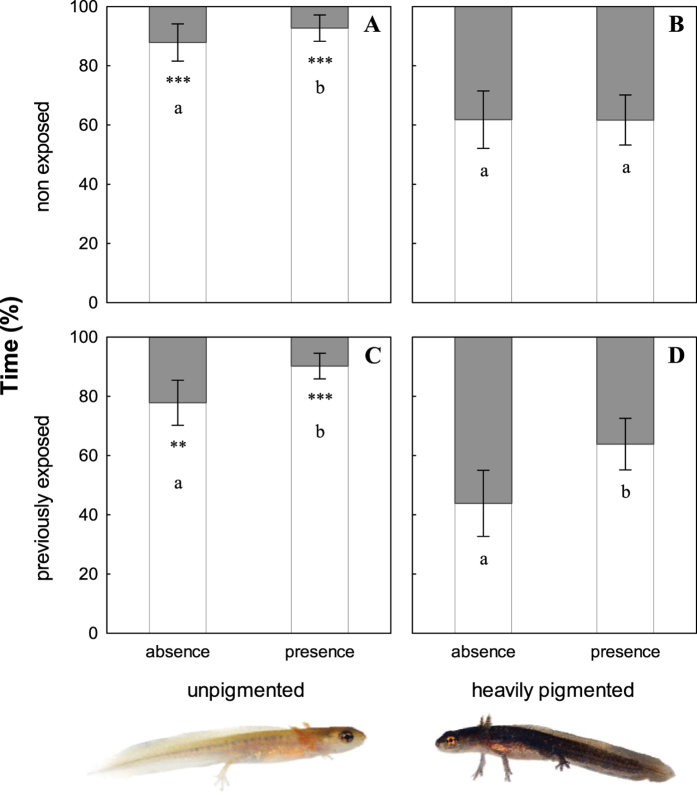
Proportion of time (mean percentage ± SE) spent in light (open bars) *vs*. dark (solid bars) environments by unpigmented and heavily pigmented larval newts subjected to light and dark microhabitat conditions respectively, after five weeks of experiment. Preferences of the same individual larva are tested in the absence and the presence of predator cues. Upper panels: larvae no previously exposed to predator cues. Bottom panels: larvae previously exposed to predator cues. Different letters indicate significant differences between testing predator cue treatments. Asterisks indicate significant preferences (**P < 0.01; ***P < 0.001).

Previous microhabitat conditions significantly affected ulterior background choice by larval newts: unpigmented larvae coming from light conditions spent more time in the light environment than heavily pigmented larvae from dark conditions, whereas the opposite occurred in the dark environment (χ^2^ = 19.14, n = 48, p < 0.0001; Fig. 3). Unpigmented larvae also spent more time in the light than in the dark environment (two-tailed binomial test, p < 0.0001), whereas heavily pigmented larvae showed no marked preference for either environment (p = 0.18; Fig. 3). Larval preferences were significantly affected by previous experimental exposure to predator cues (χ^2^ = 4.62, n = 48, p = 0.03), and by the presence or absence of predator cues during the trial (χ^2^ = 5.29, n = 48, p = 0.02). The effect of the interaction between these two factors was also significant (χ^2^ = 16.71, n = 48, p < 0.0001). Preferences of non-exposed unpigmented larvae for the light environment were stronger in the presence of predator cues (χ^2^ = 5.43, n = 11, p = 0.02; Fig. 3A), whereas testing predator cues did not influence choice behaviour in non-exposed heavily pigmented larvae (χ^2^ = 0.36, n = 11, p = 0.54; Fig. 3B). Both unpigmented and heavily pigmented newts previously exposed to predator cues increased the time spent in the light environment when predator cues were present during the trial (χ^2^ = 30.10, n = 14, p < 0.0001 and χ^2^ = 37.22, n = 12, p < 0.0001 respectively; Fig. 3C,D). In the absence of predator cues, non-exposed larvae tended to spend more time in the light environment than previously exposed larvae (χ^2^ = 2.8, n = 48, p = 0.09), whereas in the presence of predator cues, preferences of exposed and non-exposed larvae did not significantly differ (χ^2^ = 0.003, n = 47, p = 0.96) (Fig. 3). The mean time spent in the light environment by exposed and non-exposed larvae did not significantly differ within unpigmented or heavily pigmented larvae (χ^2^ = 2.39, n = 25, p = 0.12 and χ^2^ = 0.48, n = 23, p = 0.49 respectively; Fig. 3).

## Discussion

**Figure 4:**
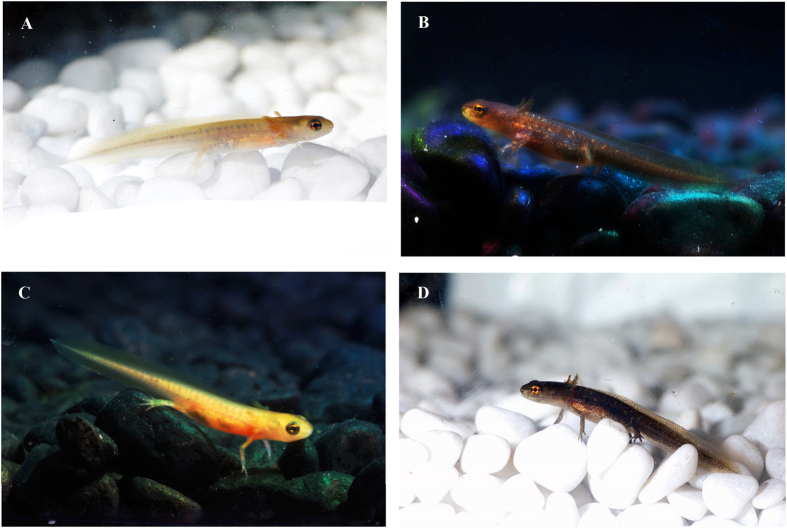
Phenotypic variation in skin pigmentation of larval newts at week 5 of the experiment. (**A**) Depigmentation induced by light microhabitat conditions, (**B**) over-pigmentation induced by dark microhabitat conditions, (**C**) microhabitat inversion: unpigmented larvae from light conditions are transferred to new dark conditions, and (**D**) microhabitat inversion: heavily pigmented larvae from dark conditions are transferred to new light conditions.

Our study revealed rapid changes in skin pigmentation of *L. boscai* larvae in response to dissimilar albedo environments. Shifts in coloration were clearly perceptible after only 3 days, and larvae maintained or even enhanced their induced phenotypes during subsequent weeks maintained in their respective light or dark microhabitats (Fig. 4). Moreover, when we inverted the microhabitat treatments, larvae responded to the new backgrounds by quickly reverting pigmentation, evidencing a remarkable ability of larval newts to achieve concealment through plastic changes in coloration in a highly reversible way. These rapid and reversible changes in coloration may constitute a key adaptation enhancing concealment in heterogeneous environments, where animals face a variety of backgrounds as they move across dissimilar habitat patches^15^.

Whereas colour change is certainly advantageous in the achievement of crypsis, it may incur in non-trivial physiological costs^26^,^23^,^24^. In amphibians and other vertebrates, pigment organelle translocations may require high energetic expenditure, since it involves complex neuroendocrine control of the chromatophores^22^,^65^,^66^. Additionally to this rapid, physiological colour change, the production of pigment particles and the number of chromatophores can be altered in response to a persistent stimulus^22^,^67^,^68^. For example, dark backgrounds are known to favour the production of melanin and inhibit the production of guanine (*i.e.*, the light-reflecting platelets), whereas light backgrounds cause the reverse effect. This slow (*i.e.*, over weeks to months), morphological colour change may also entail important metabolic costs associated to melanin synthesis or apoptosis^32^,^69^,^70^.

While the capacity of amphibians to change their colour is well known^35^,^67^,^68^,^71^, most studies have focused on anurans with special attention to post-metamorphic coloration (*e.g.* refs 72, 73, 74, but see ref. 57). In larval salamanders, changes in pigmentation have been observed mainly in *Ambystoma* sp. in response to environmental factors such as temperature and ultraviolet (UV) radiation, water turbidity and substrate colour, with degree of plasticity varying over larval ontogeny^75^,^76^,^77^,^78^. Together with protection against UV-induced damage^29^,^79^,^80^, prevention from visual predators appears to be the most relevant function of such colour plasticity in *Ambystoma* larvae and the one demonstrated here in the genus *Lissotriton*^3^,^28^,^40^,^41^ (but see ref. 81).

In our experiment, SMR in heavily pigmented larvae exposed to dark, low-reflecting microhabitats for two weeks was more than 60% higher than that of unpigmented larvae exposed to light, high-reflecting microhabitats during the same period of time. The fact that more pigmented larvae showed higher rates of oxygen consumption suggests that melanin dispersion in the dermis (physiological colour change) or an increase in the number of melanosomes and/or melanophores (morphological colour change) is physiologically costly. The existence of these production costs for pigmentation plasticity may limit camouflage in dark environments. As a consequence, prey forced to achieve crypsis in dark environments might incur higher fitness costs derived from colour change than prey in light environments.

Due in part to the observed metabolic costs of colour plasticity, the perceived level of risk may play an important role in the achievement of crypsis through colour change. In consequence, we could expect colour change to be a threat-sensitive response, with plastic organisms more readily changing their coloration in environments in which they perceive a high risk of predation. Contrary to our expectations, however, cryptic phenotypes of larval newts were not boosted by the presence of water-borne cues from predatory dragonfly nymphs, suggesting that pigmentation change improving concealment is positively selected with independence of the perceived risk of predation. Garcia and Sih^76^ also observed rapid colour change in *Ambystoma* larvae when switching from dark to light backgrounds and vice versa, but, as occurred in our experiment, the presence of chemical cues from fish predators was not found to influence background colour matching. Together, these results suggest that colour change in larval salamanders is not fine-tuned to the level of predation risk, but rather tuned to the surrounding light conditions. Since predation is a great selective force in aquatic ecosystems^82^ the consequences of suboptimal crypsis due to inaccurate assessment of predation risk might likely exceed the costs of colour plasticity^24^. Thus, this threat-independent rapid colour change is likely adaptive, since it confers the ability to speedily mimic backgrounds, increasing crypsis and preventing visual detection by predators in heterogeneous environments^15^,^28^,^75^,^83^.

In addition to colour change, antipredatory responses enhancing crypsis may also involve behavioural adaptations^15^. For example, prey animals can decrease their probability of being detected by selecting appropriate backgrounds matching their phenotype^51^,^52^,^56^. These preferences of prey might provide concealment against predators without incurring the costs of colour plasticity. Previous research in larval amphibians has pointed out a relationship between background colour and antipredator responses, with individuals selecting backgrounds enhancing their crypsis potential after being disturbed^57^, or avoiding higher activity rates in non-matching substrates^58^. Nevertheless, none of these studies have demonstrated larval ability to flexibly choose backgrounds resembling their own appearance. In our study, we found partial evidence for larvae selecting backgrounds matching their induced phenotypes. Although pigmentation induced by environmental albedo subsequently affected habitat choice by *L. boscai* –unpigmented larvae spent more time in the light environment than heavily pigmented larvae, and the opposite was true in the dark environment–, only unpigmented larvae showed a strong preference for their matching background, whereas heavily pigmented larvae showed no clear preferences at all. Moreover, heavily pigmented newts also increased the time spent in light environments in the presence of predator cues, likely waiving crypsis in favour of reaching the safer and shallower areas of the pond associated with brighter conditions. In sum, larval newts in our experiment preferred the light environment, and these preferences where stronger in the presence of predator cues (Fig. 3). Newts previously exposed to predator cues increased the time spent in light environments in the presence of cues. Predation risk is therefore a contributing factor influencing background choice in *L. boscai*.

A combination of adaptive preferences for light environments, in which camouflage presumably involves less physiological cost, together with a certain degree of imprinting onto the microhabitat conditions experienced during the previous weeks, might explain the behavioural pattern observed. Animals with colour plasticity may prefer environments where the metabolic costs of colour change are reduced, even if initially they are not well camouflaged. Garcia and Sih^76^ found evidence for colour plasticity interacting with colour-dependent antipredator behaviour in *Ambystoma* sp., in a way that species with greater capacity for colour change would undergo weaker pressures to evolve colour-dependent background choice^23^,^76^. Though often presumed, the idea that prey recognize and prefer appropriate habitats conferring crypsis based on their own phenotype is not well supported by our data. Visual complexity of the background reducing the risk of detection^84^,^85^, and/or behavioural imprinting for particular environments^86^, might alternatively explain the selection of safe backgrounds by some prey.

## Methods

### Study animals and experimental setup

We collected *L. boscai* larvae (*n* = 60) by dip-netting at several ponds in Doñana National Park, south-west Spain. Natural microhabitats of larval newts at these ponds generally consist of a grey sandy soil and bushes of hygrophytes, submerged macrophytes and floating macrophytes. Light conditions mostly depend on vegetation density and proximity to photic, shallow areas. All larvae were at the same developmental stages (stage stage 45–47, following Shi and Boucaut^87^) and were similarly sized (mean ± SD = 1.93 ± 0.28). Larvae were transported to Doñana Biological Station in Seville and housed in a walk-in climatic chamber with controlled temperature and photoperiod (20 °C; L:D 12:12). To analyse the capacity of larval newts to change skin coloration in response to different albedo environments, we used 1 L translucent plastic buckets arranged in two opposed microhabitat treatments, *i.e.*, ‘light’ *vs*. ‘dark’. Buckets in the ‘light’, high-reflecting treatment consisted of a substrate of white gravel at the bottom and a white plastic sleeve covering the walls. Buckets in the ‘dark’, low-reflecting treatment were provided with a black gravel substrate and covered with black plastic sleeves in identical manner. Buckets were filled with carbon-filtered dechlorinated tap water and received one larva each. Water was renewed twice weekly and newts were fed mosquito larvae every other day. We randomly assigned half of the larvae to the ‘light’ treatment and the other half to the ‘dark’ treatment (*n* = 30 each). To analyse the potential reversibility of inducible changes in coloration, microhabitat treatments were inverted after five weeks of experiment, so that larvae in the ‘light’ treatment were transferred to the ‘dark’ treatment and vice versa. From this moment onwards, we continued the experiment for two additional weeks (until week 7). By the end of the experiment some of the initial larvae had already metamorphosed and a few died, so we tested reversibility on a reduced sample (*n* = 41).

To test for the effect of predator cues on inducible changes in skin coloration of larval newts, we also dip-netted dragonfly nymphs (*A. imperator*) at several ponds within the Park, to be used as predator cue donors. Dragonflies were also housed in the climatic chamber and kept individually in 1 L plastic buckets. To prepare predator chemical cues, we filled each donor dragonfly aquarium with 0.5 L of dechlorinated tap water, to be pervaded with predator cues. Since these cues last c. 2–4 days in water^88^, we extracted and mixed the water from three donor aquaria every 48 h. To prepare control water we followed the same procedure but without placing predators in the aquaria^89^,^90^,^91^. For each microhabitat treatment, we randomly assigned half of the larvae to a ‘predator’ treatment and the other half to a ‘non-predator’ treatment, setting a 2 × 2 factorial design (light *vs*. dark x predator presence *vs*. absence; *n* = 15 in each treatment combination). Every 48 h, 10 mL of water containing predator cues were added to buckets assigned to the ‘predator’ treatment, whereas buckets assigned to the ‘non-predator’ treatment received 10 mL of control water. Similar volumes of predator cues added to water are known to systematically induce antipredator behavior in amphibian larvae^91^,^92^,^93^. Dragonflies were fed anuran tadpoles from a stock tank, once per day. At the end of the experiments, all surviving larval and metamorphic newts were released at their ponds of origin after standard prophylaxis procedures, whereas no dragonflies survived. All experiments were carried out in accordance with all current European directives and Spanish laws, and under permission of the Consejería de Medio Ambiente from Junta de Andalucía. Procedures conformed to the recommended guidelines for use of live amphibians and reptiles in laboratory research^94^. All experimental protocols were approved by the ‘Comité de Ética de Experimentación Animal CEEA-EBD’.

### Image processing

Induced changes in skin pigmentation of larval newts were assessed through quantitative image analysis. A side-view digital image of each larva was taken before the experiment began and then at different moments throughout the experiment (*i.e.*, day 0, day 3, week 2, week 5, and week 7). Images were processed using Adobe Photoshop CS3 Extended, blindly to information about treatments and time of the experiment. We followed the methodological approach of Mayani-Parás *et al*.^95^, who estimated avian egg camouflage by quantifying the proportion of eggshell covered with dirt. For each picture, we selected a standardized area (1 × 3 mm) covering the central part of the animal body between the anterior and posterior limbs. Then, we carefully selected every dark pixel within this area, using the “magic wand” tool and refining the selection by picking up pixels discretely. Percentage of skin pigmentation was calculated as the relationship between the number of pixels selected and the total number of pixels in the standardized area. This way, we obtained an accurate measure of pigmentation for each individual larva at different moments in the experiment. We used this measure to compare skin pigmentation of larval newts across microhabitat and predator cue treatments, and within the same individual larva throughout the course of the experiment.

### Metabolic cost

To measure SMR on newt larvae, we used a flow-through aquatic respirometer consisting of five cylindrical chambers (44 mm diameter × 163 mm long) that were supplied with oxygen-saturated water from a header tank at a constant flow. Ten optical sensors (optodes; Oxy 10-PreSens, Germany) were mounted at the entrance and exit of the chambers and connected to an oxymeter (Oxy 10-PreSens, Germany) that recorded oxygen concentration every 15 seconds. This way we simultaneously obtained measures of oxygen consumption (mg/L) for five independent individuals. The respirometer was calibrated at least once daily using a saturated sodium sulphite solution and oxygen saturated water to achieve 0 and 100% oxygen concentrations^96^. Respirometry trials were conducted after two weeks of experiment, when changes in skin pigmentation were clearly observable. All the measurements were taken at 20 °C, the same temperature experienced by larvae in the walk-in climatic chamber during the previous weeks. For the trials, larvae (*n* = 47) were introduced individually in the chambers. Not all the initial larvae could be tested because some metamorphosed and a few died during the course of the experiment. Thus, the sample distribution across treatment was as follows: light x predator *n* = 13; light x non-predator *n* = 12; dark x predator *n* = 13; dark × non predator *n* = 9. We allowed larvae to acclimate in the chambers for five minutes and then we recorded oxygen consumption in each chamber for 30 min. SMR was calculated as:


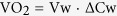


where VO_2_ is the rate of oxygen consumption (μg h^−1^), Vw is the flow rate of water through the chamber (L h^−1^), and ΔCw is the instantaneous difference in oxygen concentration between the inflow and outflow^96^,^97^. After the trials, each larva was blotted dry and weighed on a high precision balance (CP324S, Sartorius, precision: ±0.1 mg), for inclusion of body mass as a covariate in statistical analyses.

### Background choice behaviour

In a subsequent experiment, we compared preferences for light *vs*. dark environments of larval newts that had been previously subjected to light or dark microhabitat conditions, in the presence and absence of predator cues. These behavioural assays took place after five weeks of experiment, right before inverting microhabitat conditions. Larvae were tested in transparent aquaria (32 × 17 × 18 cm, 10 L) that were divided in two spaces of equal surface and volume, but offering two opposed microhabitat conditions, light *vs*. dark. The light side of each experimental aquarium was provided with a white gravel substrate and we covered the walls with a white plastic sleeve, whereas the dark side was provided with a black gravel substrate and the walls were covered with a dark plastic sleeve. There was no physical separation between the two sides, so that larvae were free to move from one environment to the other. We tested each individual larva (*n* = 48) in two predator cue treatments (‘presence’ *vs*. ‘absence’) in a random sequence. In the ‘presence’ treatment, we added to the aquaria 20 mL of water pervaded with predator cues from three dragonfly nymphs, whereas in the ‘absence’ treatment we added 20 mL of control water. Larvae were given 24 h between trials to rest. The position of the light and the dark environments in experimental aquaria was randomized across trials and treatments.

At the beginning of the trials, we placed a single larva in the middle of each experimental aquarium, so it was given the choice between the two environments. We waited 5 min before the trials began to allow larvae to acclimate, and then we monitored each larva for 30 min, using the instantaneous scan sampling method, and recording every 1 min the side that each larva occupied in the aquarium (30 scans per larva in total). Preferences of larvae for light *vs*. dark environments were assessed from the proportion of scans they spent in each side of the aquarium. Then, we used this measure to estimate the effects of previous microhabitat conditions (‘light’ *vs*. ‘dark’) and previous exposure to predator cues (‘predator’ *vs*. ‘non-predator’) on larval preferences for light *vs*. dark environments in the presence or absence of predator cues.

### Data analyses

To analyse changes in skin coloration of larval newts induced by microhabitat conditions or exposure to predator cues, we used a linear mixed model (LMM) with time of the experiment (four levels: ‘day 0’, ‘day 3’, ‘week 2’ or ‘week 5’), microhabitat (‘ligh’ *vs*. ‘dark’) and predator cue (‘predator’ *vs*. ‘non-predator’) as three independent factors, individual larva as random factor, and percentage of skin pigmentation as dependent variable. To analyse the reversibility of inducible changes in pigmentation, we used a LMM with time of the experiment (two levels: ‘week 5’ *vs*. ‘week 7’), microhabitat (‘light’ *vs*. ‘dark’) and predator cue (‘predator’ *vs*. ‘non-predator’) as three independent factors, individual larva as random factor, and percentage of skin pigmentation as dependent variable. Percentage of skin pigmentation was non-normally distributed and was transformed using Box-Cox power transformations. Post-hoc comparisons among treatments were made using Tukey’s honestly significant difference tests^98^.

To analyse differences in SMR of larval newts associated to skin pigmentation we used a forward stepwise general regression model (GRM) with microhabitat (‘light’ *vs*. ‘dark’) and predator cue (‘predator’ *vs*. ‘non-predator’) treatments as two categorical independent predictors, weight and percentage of pigmented skin (week 2) as two continuous independent predictors, and SMR as dependent variable. To analyse background choice behaviour by larval newts we used a generalized linear mixed model (GLMM) with a binomial distribution. Microhabitat (‘light’ *vs*. ‘dark’), predator cue (‘predator’ *vs*. ‘non-predator’) and testing predator cue (‘presence’ *vs*. ‘absence’) were included as three independent factors, individual larva as random factor, and percentage of time spent in light *vs*. dark environments as dependent variable. All analyses were performed in Statistica 12.0 and R 3.1.3.

## Additional Information

**How to cite this article**: Polo-Cavia, N. and Gomez-Mestre, I. Pigmentation plasticity enhances crypsis in larval newts: associated metabolic cost and background choice behaviour. *Sci. Rep.* **7**, 39739; doi: 10.1038/srep39739 (2017).

**Publisher's note:** Springer Nature remains neutral with regard to jurisdictional claims in published maps and institutional affiliations.
